# Severe neurological complications of leptospirosis, presentation of two cases

**DOI:** 10.1016/j.idcr.2026.e02505

**Published:** 2026-01-24

**Authors:** Stéphanie M.L.M. Looijaard, Hetty Jolink, Nathalie M. Delfos, Fanny N. Lauw

**Affiliations:** a Department of Internal Medicine, Alrijne hospital, Leiderdorp, the Netherlands; b Department of Infectious diseases, Leiden University Medical Center, Leiden, the Netherlands

**Keywords:** Leptospirosis, Nervous system diseases, Polyradiculopathy, Intracranial hemorrhages, Corticosteroids, Case report

## Abstract

We discuss two cases of severe leptospirosis, in which the most concerning symptoms affected the nervous system. These cases illustrate the wide range of neurological symptoms that can be seen in patients with leptospirosis, which can affect both the central and peripheral nervous system. These symptoms are thought to be caused by an inflammatory reaction to *Leptospira*, rather than by the infection itself. Both patients experienced a variety of neurological symptoms that prolonged and altered their course of treatment. The first patient developed radicular pain secondary to polyradiculitis, which was confirmed by spinal MRI. He was treated with antibiotics but continued to experience bilateral leg pain following treatment. He was referred to a rehabilitation clinic to help him deal with his persisting complaints. The second patient was admitted to the intensive care unit and failed to regain consciousness after sedation was discontinued. Neuroimaging revealed multiple intracranial microhemorrhages. He was treated with antibiotics in combination with corticosteroids. Following extensive rehabilitation, he recovered without residual neurological deficits.

## Highlights

• Patients with leptospirosis can develop a range of neurological symptoms.
• Inflammatory response can cause a cytokine storm, and lead to severe complications.
• These include polyradiculitis and intracranial microhemorrhages.
• Corticosteroids might be indicated in immune-mediated symptoms and improve outcome.

## Introduction

Leptospirosis is a zoonotic infection transmitted via direct or indirect exposure to contaminated soil or freshwater polluted with urine of rodents or other mammals infected with pathogenic *Leptospira* species. Although leptospirosis is most prevalent in tropical and subtropical regions, the disease affects animals and humans worldwide [1], [2]. Global climate change is expected to further increase human exposure to pathogenic *Leptospira* species, largely due to more frequent flooding events and the accumulation of stagnant water [3]. In addition, ongoing globalization, which has led to increased international travel, can further heighten the risk of worldwide outbreaks [1]. Based on historic data, the most recent estimates report an annual incidence of more than one million cases worldwide, with substantial mortality numbers in those with severe illness [2]. These numbers highlight the global burden of leptospirosis and its consequences for public health, the economy, and overall disease burden [4], [5].

The typical course of leptospirosis includes an initial acute phase with mild symptoms such as fever, myalgia, and headache [6]. Leptospirosis can progress to a severe form with multi-organ failure and hemorrhage [6]. The best-known example of severe leptospirosis is also known as Weil’s disease, classically presenting with a combination of jaundice, kidney failure, and hemorrhage [7]. After resolution of the acute phase, most patients gradually recover [8]. Some patients, however, develop a second phase, also known as the immune phase, which is characterized by high circulating levels of pro-inflammatory cytokines such as interleukin-1 beta (IL-1β), interleukin-6 (IL-6), and tumor necrosis factor alpha (TNF- α) [6], [9]. The high levels of circulating cytokines are associated with more severe manifestations of leptospirosis [6], [9]. The inflammatory response can ultimately cause a cytokine storm, leading to severe complications such as pulmonary hemorrhage and multi-organ failure in patients with leptospirosis [6], [8], [9].

In this article, we present two uncommon presentations of immune-mediated neurological symptoms in patients with leptospirosis and elaborate on the role of pro-inflammatory cytokines and the role of corticosteroid treatment.

## Case presentation

The first patient was a 33-year-old man who presented at the emergency department (ED) with a five-day history of myalgia, arthralgia, and fever. He had dived into freshwater 18 days earlier while celebrating the New Year. At the ED, he required a wheelchair because of generalized pain. Physical and neurological examinations revealed no abnormalities. Laboratory results showed elevated inflammatory markers, thrombocytopenia, acute kidney failure, abnormal liver function, and elevated creatinine kinase (Table 1). Urinalysis, testing for respiratory viruses, and chest X-ray were unremarkable. The patient was admitted with suspected leptospirosis based on the presence of myalgia and fever, in combination with laboratory findings and recent freshwater exposure. He was treated empirically with intravenous benzylpenicillin. The next day, he developed severe headache and petechial hemorrhages. Cerebrospinal fluid analysis was consistent with an aseptic meningitis, showing an increased total cell count, elevated protein levels, and regular glucose levels [10]. Over the subsequent days, real-time polymerase chain reaction (qPCR) targeting the *lipL32* gene [11] detected *Leptospira* DNA in serum, urine, and cerebrospinal fluid. The patient completed a seven-day course of antibiotics and was discharged in a clinically improving condition. In addition, serologic testing was performed, and microscopic agglutination test results indicated *Leptospira icterohaemorrhagiae* as the likely causative serogroup.

**Table 1:** Laboratory results at time of admission.

**Laboratory marker**	**Patient #1**	**Reference range**	**Patient #2**	**Reference range**
Erythrocyte sedimentation rate (mm/h)	31	1–10	53	0–20
Hemoglobin (g/dL)	13.2	13.7–17.7	14.7	13.7–17.7
Thrombocytes (10^9/L)	42	150–400	18	150–400
Leukocytes (10^9/L)	11.5	4.0–10.0	8.3	4.0–10.0
C-reactive Protein (mg/L)	201	0–5	403	< 5
Creatinine (µmol/L)	299	64–104	204	64–104
Sodium (mmol/L)	128	135–145	138	136–145
Bilirubin total (µmol/L)	49	3–20	45	< 17
Bilirubin conjugated (µmol/L)	28	0–4	32	< 3
Alkaline phosphatase (U/L)	156	0–115	104	< 115
Gamma-glutamyltransferase (U/L)	168	0–55	36	< 55
Aspartate-aminotransferase (U/L)	170	0–35	44	< 35
Alamine-aminotransferase (U/L)	154	0–45	29	< 45
Lactate dehydrogenase (U/L)	315	0–248	193	< 248
Creatine kinase (U/L)	895	0–171	569	< 171

Approximately one week later, the patient developed radicular pain, accompanied by weakness and sensory symptoms in both legs. He was evaluated at the neurology outpatient clinic, where magnetic resonance imaging (MRI) showed findings consistent with a polyradiculitis/adhesive arachnoiditis. He was readmitted to the neurology department, and qPCR tests on serum, urine, and cerebrospinal fluid were repeated. Due to concern for ongoing *Leptospira* infection, treatment with high-dose benzylpenicillin was initiated pending microbiology results. Antibiotics were given for five days, after which he was discharged. qPCR testing remained negative for *Leptospira*, supporting an inflammatory response as the cause of his symptoms. Follow-up showed normalized laboratory values, but the patient experienced persistent bilateral leg pain, which impacted his daily activities. A repeat MRI of the lumbar spine confirmed findings consistent with adhesive arachnoiditis, without signs of active inflammation. He was referred to a rehabilitation clinic for further management of his persistent symptoms.

The second patient was a 60-year-old man, who presented at the ED with a septic shock, headache, and myalgia. He had a four-day history of high fevers, mild abdominal pain, and frequent loose stools. One week before presentation he had gone windsurfing at a lake. At the ED, we saw a severely ill patient with conjunctival suffusion, without any neurological signs or symptoms. Laboratory results showed elevated inflammatory markers, thrombocytopenia, acute renal insufficiency, elevated creatinine kinase, and signs of disseminated intravascular coagulation (Table 1). Urinalysis and chest X-ray showed no abnormalities. He was admitted to the intensive care unit (ICU) and treated with ceftriaxone for suspected leptospirosis based on the combination of fever, myalgia, conjunctival suffusion, laboratory findings, and recent freshwater exposure. Ceftriaxone was changed to benzylpenicillin upon confirmation of the diagnosis leptospirosis, with a positive *Leptospira* qPCR targeting the *lipL32* gene [11] on serum and urine. Microscopic agglutination test was conducted, but could not identify the likely causative serogroup. Additional PCR-based typing did establish *Leptospira interrogans* infection.

Despite vasopressors, the patient hemodynamically deteriorated. Because of exhaustion there was an indication for mechanical ventilation. After four days he improved and sedation could be stopped. Unfortunately, the patient remained comatose two days after complete cessation of the sedative medication. An MRI of the brain showed diffuse multiple intracranial microhemorrhages, compatible with leptospirosis-associated vasculopathy. After extensive multidisciplinary consultation, it was decided to treat with methylprednisolone, followed by prednisolone taper. He slowly improved neurologically, and after four weeks he was discharged from the ICU. He experienced several hospital-acquired infections in the following weeks, including a hospital-acquired pneumonia and a urinary-tract infection. After a hospital stay of 2.5 months he was discharged to a rehabilitation clinic. Eventually he recovered completely without neurological sequelae.

## Discussion

Patients with leptospirosis can experience a wide spectrum of clinical symptoms during the course of infection [6], as well as during the inflammatory period that can follow [8], [9]. Neurological manifestations in patients with leptospirosis occur but available literature is limited to case reports. Both presented patients in this case report demonstrate that when patients with leptospirosis develop new symptoms after the initial presentation or apparent recovery, it is crucial to determine whether these manifestations are due to a persistent infection or inflammatory processes. Usually, the infectious phase of leptospirosis should improve during antibiotic treatment and resolve within approximately one week. The immune phase that can follow, is accompanied by high levels of circulating cytokines and can lead to a wide range of symptoms [6], [8], [9]. Pro-inflammatory cytokines such as IL-1β, TNF-α, interferon-γ, and IL-6 can enhance blood-brain barrier permeability by disrupting tight junction integrity [12]. This can facilitate the entry of neurotoxic substances and peripheral immune cells into the central nervous system. The resulting neuroinflammation can lead to tissue damage and neurological symptoms in patients [12].

Immune-mediated processes are not expected to respond to additional antibiotic treatment, as their underlying mechanisms are driven by the host immune response, rather than ongoing bacterial infection. There may be a beneficial effect of anti-inflammatory drugs such as corticosteroids. Although in a different setting, dexamethasone has been shown to reduce the mortality in patients with severe Covid-19 infection by reducing overstimulation of the immune response [13]. One observational study showed that dexamethasone was associated with a lower frequency of neurological complications such as stroke, meningitis and encephalitis, in patients with severe Covid-19 [14]. In the first case presented, treatment with corticosteroids was not administered, while antibiotic treatment was repeated because of concern for ongoing infection. The benefit of repeated antibiotic treatment in the absence of circulating *Leptospira,* is highly questionable. It is unknown whether corticosteroids would have had a beneficial effect on the course of the patient’s symptoms. In the second case, corticosteroids were initiated and the patient slowly recovered afterwards.

The different treatment approaches in these cases raises the question whether there is evidence supporting the use of corticosteroids in immune-mediated neurological manifestations in patients with leptospirosis. Most research in this field has been conducted in patients with leptospirosis and pulmonary involvement. A recent meta-analysis on this topic [15] concluded that four out of five studies [16], [17], [18], [19] showed potentially favorable results on clinical outcome with a lower risk of mortality in patients treated with corticosteroids. However, this effect did not reach statistical significance, was particularly shown in patients with lung involvement and the methodological quality of the studies was poor [15]. The only randomized controlled trial that was included did not show a beneficial effect [20] and overall, an increased risk of nosocomial infections was seen in patients receiving corticosteroids [15]. Another recent meta-analysis including some of the same studies, could not confirm an effect of corticosteroids on mortality in patients with leptospirosis either [21]. Only one case series has been found that specifically discusses the use of corticosteroids in patients with leptospirosis and neurological involvement [22]. These cases concerned children with an encephalomyelitis and suggested a beneficial effect of corticosteroids [22]. Given the limited data on the use of corticosteroids for immune-mediated neurological involvement in patients with leptospirosis, it is recommended to assess this decision on an individual basis for each patient and discuss it within a multidisciplinary team. When antibiotic treatment seems to have efficiently eradicated any circulating *Leptospira* species, subsequent manifestations are likely due to inflammatory processes and may benefit from corticosteroids.

In conclusion, the presented cases illustrate uncommon and severe neurological presentations due to immune-mediated processes in patients with leptospirosis. Adjunctive treatments beyond antibiotics, such as corticosteroids targeting the immune response, may improve patient outcomes. Due to the paucity of data, the use of corticosteroids should be assessed individually in collaboration with a multidisciplinary team.

## Ethical approval

No ethical approval was required for this manuscript.

## Consent

Written informed consent was obtained from the patient for publication of this case report and accompanying images. A copy of the written consent is available for review by the Editor-in-Chief of this journal on request.

## Funding

This research did not receive any specific grant from funding agencies in the public, commercial, or not-for-profit sectors.

## CRediT authorship contribution statement

**Fanny N. Lauw:** Writing – review & editing, Writing – original draft, Conceptualization. **Stephanie M.L.M. Looijaard:** Writing – review & editing, Writing – original draft, Data curation, Conceptualization. **Hetty Jolink:** Writing – review & editing, Writing – original draft, Data curation, Conceptualization. **Nathalie M. Delfos:** Writing – review & editing, Conceptualization.

## Declaration of Competing Interest

The authors declare that they have no known competing financial interests or personal relationships that could have appeared to influence the work reported in this paper.
